# The ABA-induced soybean ERF transcription factor gene *GmERF75* plays a role in enhancing osmotic stress tolerance in *Arabidopsis* and soybean

**DOI:** 10.1186/s12870-019-2066-6

**Published:** 2019-11-20

**Authors:** Meng-Jie Zhao, Li-Juan Yin, Ying Liu, Jian Ma, Jia-Cheng Zheng, Jin-Hao Lan, Jin-Dong Fu, Ming Chen, Zhao-Shi Xu, You-Zhi Ma

**Affiliations:** 1 Institute of Crop Sciences, Chinese Academy of Agricultural Sciences (CAAS)/National Key Facility for Crop Gene Resources and Genetic Improvement, Key Laboratory of Biology and Genetic Improvement of Triticeae Crops, Ministry of Agriculture, Beijing, 100081 China; 2 Faculty of Agronomy, Jilin Agricultural University, Changchun, 130118 China; 3 Anhui Science and Technology University, Fengyang, 233100 China; 4 College of Agronomy, Qingdao Agricultural University, Qingdao, 266109 China

**Keywords:** Ethylene-responsive factor, Hypocotyl elongation, Root growth, Response mechanism, Osmotic tolerance, Soybean

## Abstract

**Background:**

Ethylene-responsive factors (ERFs) play important roles in plant growth and development and the response to adverse environmental factors, including abiotic and biotic stresses.

**Results:**

In the present study, we identified 160 soybean ERF genes distributed across 20 chromosomes that could be clustered into eight groups based on phylogenetic relationships. A highly ABA-responsive ERF gene, *GmERF75*, belonging to Group VII was further characterized. Subcellular localization analysis showed that the GmERF75 protein is localized in the nucleus, and qRT-PCR results showed that *GmERF75* is responsive to multiple abiotic stresses and exogenous hormones. *GmERF75*-overexpressing *Arabidopsis* lines showed higher chlorophyll content compared to WT and mutants under osmotic stress. Two independent *Arabidopsis* mutations of *AtERF71*, a gene homologous to *GmERF75*, displayed shorter hypocotyls, and overexpression of *GmERF75* in these mutants could rescue the short hypocotyl phenotypes. Overexpressing *GmERF75* in soybean hairy roots improved root growth under exogenous ABA and salt stress.

**Conclusions:**

These results suggested that *GmERF75* is an important plant transcription factor that plays a critical role in enhancing osmotic tolerance in both *Arabidopsis* and soybean.

## Background

Plants have a complex and elaborate regulation mechanism to defense the environmental factors including abiotic and biotic stresses [1, 2]. Transcription factors, regulators of genes expression, perform pivotal functions in signal transduction networks where they directly activate or suppress targeted genes expression so that the interaction between different signaling pathways was impacted [3–5].

APETALA2/Ethylene Responsive Factor (AP2/ERF) superfamily, a large gene family in plant, play important roles in signal transduction, plant growth and development, and involved in biotic and abiotic stresses response [6]. According to its conservative domain, AP2/ERF can be divided into three major families: APETALA2(AP2), Ethylene Responsive Factor (ERF), and RELATED TO ABSCISIC ACID INSENSITIVE 3/VIVIPAROUS 1 (RAV) [7]. The AP2 family contains two AP2/ERF domain, ERF family which can be divided into two subfamilies: DEHYDRATION-RESPONSIVE ELEMENT BINDING proteins (DREBs) and ERFs, contains an AP2/ERF domain, and RAV family contains an AP2/ERF domain [7]. ERFs play diverse roles in plants throughout different development stage, such as seed germination, tissue formation, flower stage, response to biotic and abiotic stresses [8, 9]. Previous study found that ERFs could specifically bind to the GCC-box and/or dehydration-responsive element/C-repeat (DRE/CRT) *cis*-acting elements to regulate the downstream gene expression, such as ethylene (ET)-inducible pathogenesis-related (PR) genes and abiotic stresses-inducible genes [10]. .In recent years, it was found that ERFs could also bind to Coupling Element 1 (CE1: TGCCACCG), Hypoxia-Responsive Promoter Element (HRPE), and ATCTA [11, 12]. ERFs, were first identified in tobacco, since then more and more ERFs have been identified in diverse plants, including *Arabidopsis*, rice, *Atriplex canescens*, peanuts, sunflower, and potato [7, 13–18].

ERF could influence the growth and development in plant. The activity of some ERFs was impacted by different development stage [19]. Overexpressing *LkAP2L2* in *Arabidopsis*, which could affect seed growth, branch, flower development, and siliques, significantly enhanced the number of shoot branches and decreased the length of siliques, the number of seeds, the size and number of transgenic rosette leaves [8]. *OsHL6*, an AP2/ERF transcript factor in rice, could regulate the expression of some auxin-related genes by interacting with OsWOX3 and play critical roles in trichome formation [9].

ERF genes can also function in abiotic and/or biotic stress responsive pathways. *TaERF1*, a wheat ERF gene which could be induced by multiple environmental stresses including drought, salt, low temperature, and exogenous hormones such as ABA, ET, and salicylic acid (SA), was also identified as a defense gene against pathogen (*Blumeria graminis* f. sp. tritici). Overexpression of *TaERF1* in *Arabidopsis* and tobacco could improve resistance to pathogens and enhance tolerance to multiple abiotic stresses [20]. *Haynaldia villosa ERF1-V* regulated the response to both powdery mildew and drought and salinity when overexpressed in wheat [21]. Similarly, *TaPIE1*, a member of ERF family in wheat, enhanced resistance to *Rhizoctonia cerealis* and increased tolerance to freezing stress by activating defense- and stress-related genes that function downstream of the ET signaling pathway in wheat [22]. Therefore, ERF genes could encode multifunctional factors that respond to multiple stresses, integrate potentially various signal transduction pathways, and thus play dual roles in both abiotic and biotic stress responses in plants [14, 23].

Although ERFs have been found in diverse plants, many soybean ERFs have not been reported yet, which is one of the most economically important crop species. In addition, the functions of most ERF genes have yet to be determined. In this study, we searched for and integrated all non-redundant sets of soybean ERF genes. *GmERF75*, a highly ABA-induced ERF gene, was chosen for further expression and functional analysis. *GmERF75* was up-regulated by multiple abiotic stresses and exogenous hormones, and overexpression of which could enhance osmotic tolerance in both *Arabidopsis* and soybean.

## Results

### Identification and physical locations of soybean ERFs

**Fig. 1:**
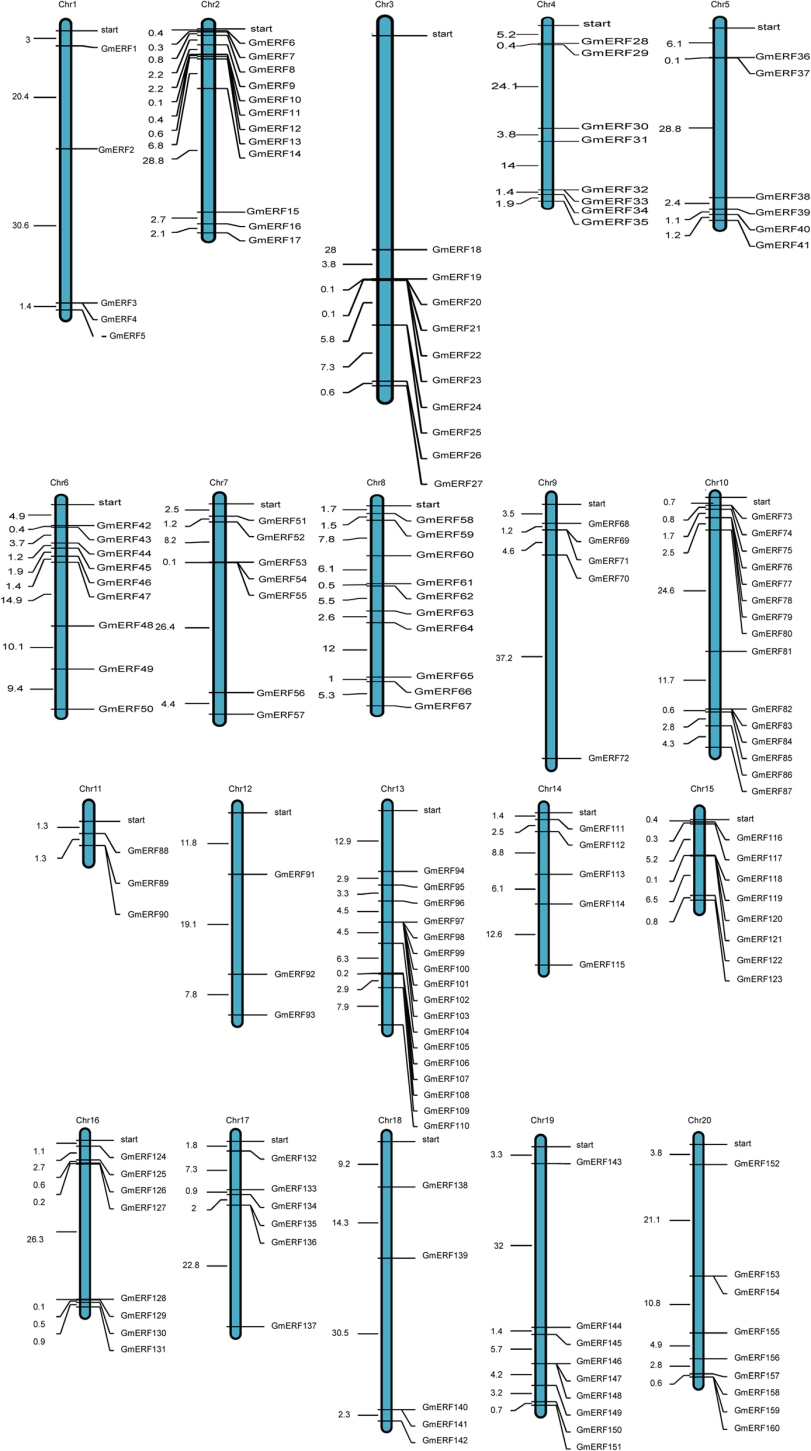
Distribution of ERF genes in the soybean genome. The blue bars represent the chromosomes (not drawn to scale), and the chromosome numbers are shown above the bars. Soybean ERFs were distributed on all 20 chromosomes. The numbers to the left of the chromosomes indicate the distances between the neighboring genes in megabases (Mb)

**Fig. 2:**
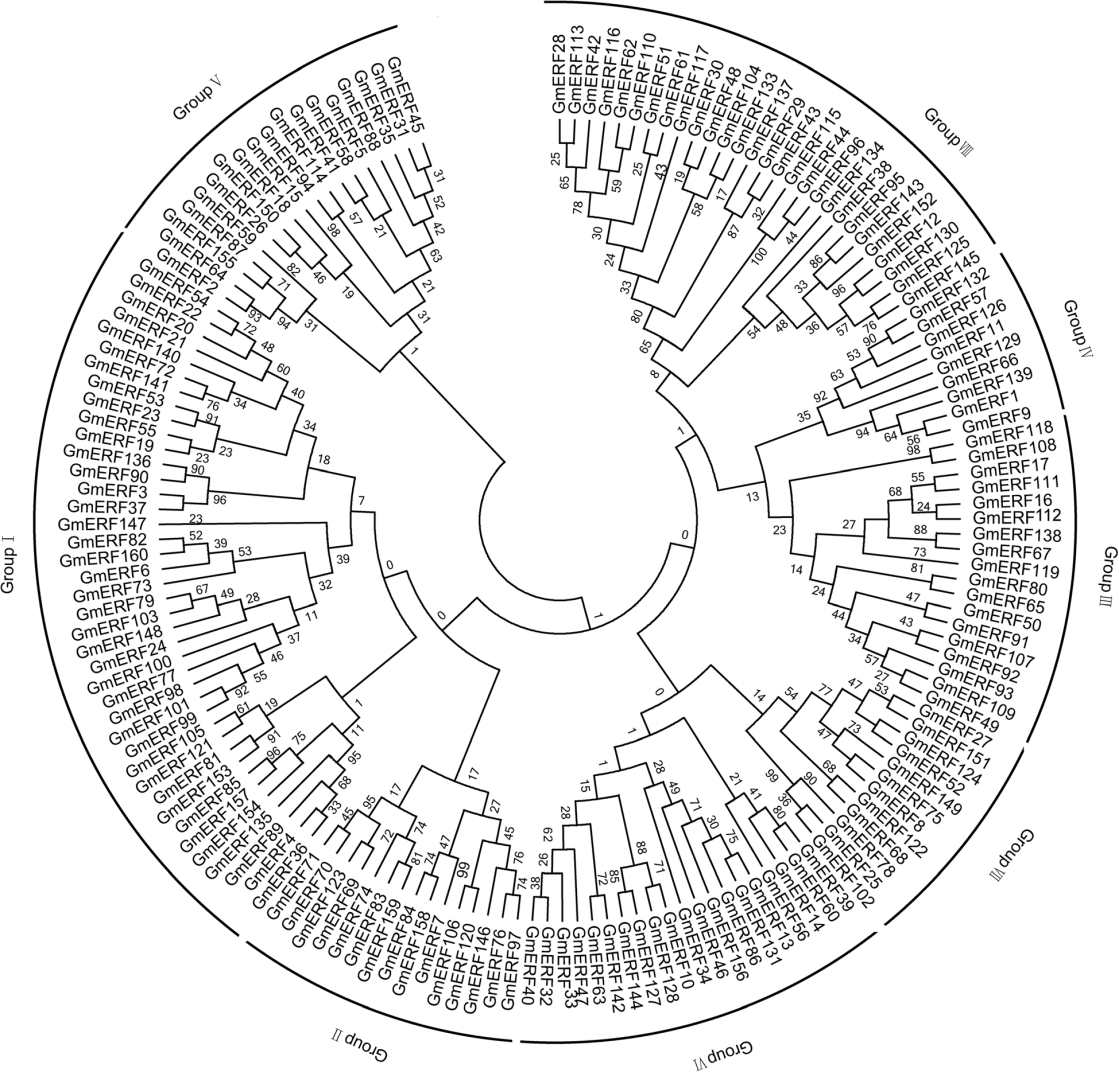
Unrooted phylogenetic tree of the soybean ERF proteins. The amino acid sequences of the AP2/ERF domains of 160 soybean ERF family proteins were aligned using ClustalW, and the phylogenetic tree was constructed using the neighbor-joining method in MEGA 5.0 (Additional file 1: Table S1)

We used the Pfam [24] and SMART databases [25] as references for the identification of 160 non-redundant soybean ERFs (Additional file 1: Table S1). According to the soybean genome database, 160 soybean ERFs were distributed across 20 chromosomes (Fig. 1). The number of ERF genes on each chromosome differed considerably. There were 17 ERF genes distributed in chromosome 13, but only 3 in chromosome 12 (Fig. 1). Multiple alignments of full-length amino acid sequences were performed using MEGA 5.1 [26] . The ERF proteins could be clustered into eight groups (I to VIII) based on their phylogenetic relationships (Fig. 2). Almost one-fourth of the ERF proteins were clustered in Group I, while only nine were clustered in Group IV.

### Expression profiles of soybean ERFs

To examine the expression patterns of ERFs, a map of soybean ERF gene expression in 14 soybean tissues and organs at different developmental stages was drawn based on the gene-chip data downloaded from the soybean genome database (Additional file 2: Figure S1; Additional file 3: Table S2). Soybean ERFs were expressed at the highest levels in the nodules of 21 days-old plants and at the lowest levels in seeds. A few soybean ERFs displayed different tissue-specific expression patterns. For example, eight ERFs were expressed in only one tissue, and nine ERFs were expressed in only two tissues. The expression levels for genes in different groups also differed. The expression levels of Group II genes were lower than those of genes in the other groups. The expression patterns of ERFs within the same group also varied. For example, *GmERF127* transcripts reached the maximum level in flowers, whereas *GmERF10* transcripts reached the highest level in roots. *GmERF6*, *GmERF66*, and *GmERF84* were expressed at a low level, whereas *GmERF52*, *GmERF112*, *GmERF122*, and *GmERF124* were expressed at an extremely high level. Interestingly, three-quarters of the extremely high-expressed ERF genes were clustered in Group VII. Therefore, Group VII was selected for further investigation.

### Conserved protein motifs and gene structures of soybean group VII ERFs

There are 12 ERF genes belonging to Group VII. To investigate the modular structure of the proteins encoded by these genes, DOG 2.0 was used to draw the domains in each protein. As shown in Additional file 4: Figure S2, each Group VII ERF protein had a typical AP2/ERF DNA-binding domain, which is highly conserved, consists of 57–61 amino acids, and contains three β-sheet regions and an α-helix. The key amino acid residues determining DNA-binding specificity are those at positions 14, Ala (A) and 19, Asp (D) [10].

Gene structure analysis was done to compare the distribution of introns and exons in each soybean ERF gene. Almost all the ERF genes contained one intron except for *GmERF102*, *GmERF25*, and *GmERF78* which contained no intron (Additional file 5: Figure S3).

### Expression pattern of *GmERF75* under ABA treatment

ABA plays essential role in regulating seed germination, growth and development, and responses to environmental stresses in plants [10, 27, 28]. It has been reported that most drought-inducible and/or salt-inducible genes were also induced by exogenous ABA treatment in *Arabidopsis* [29], which suggested the existence of cross-talk between ABA and osmotic stress signaling pathways.

**Fig. 3:**
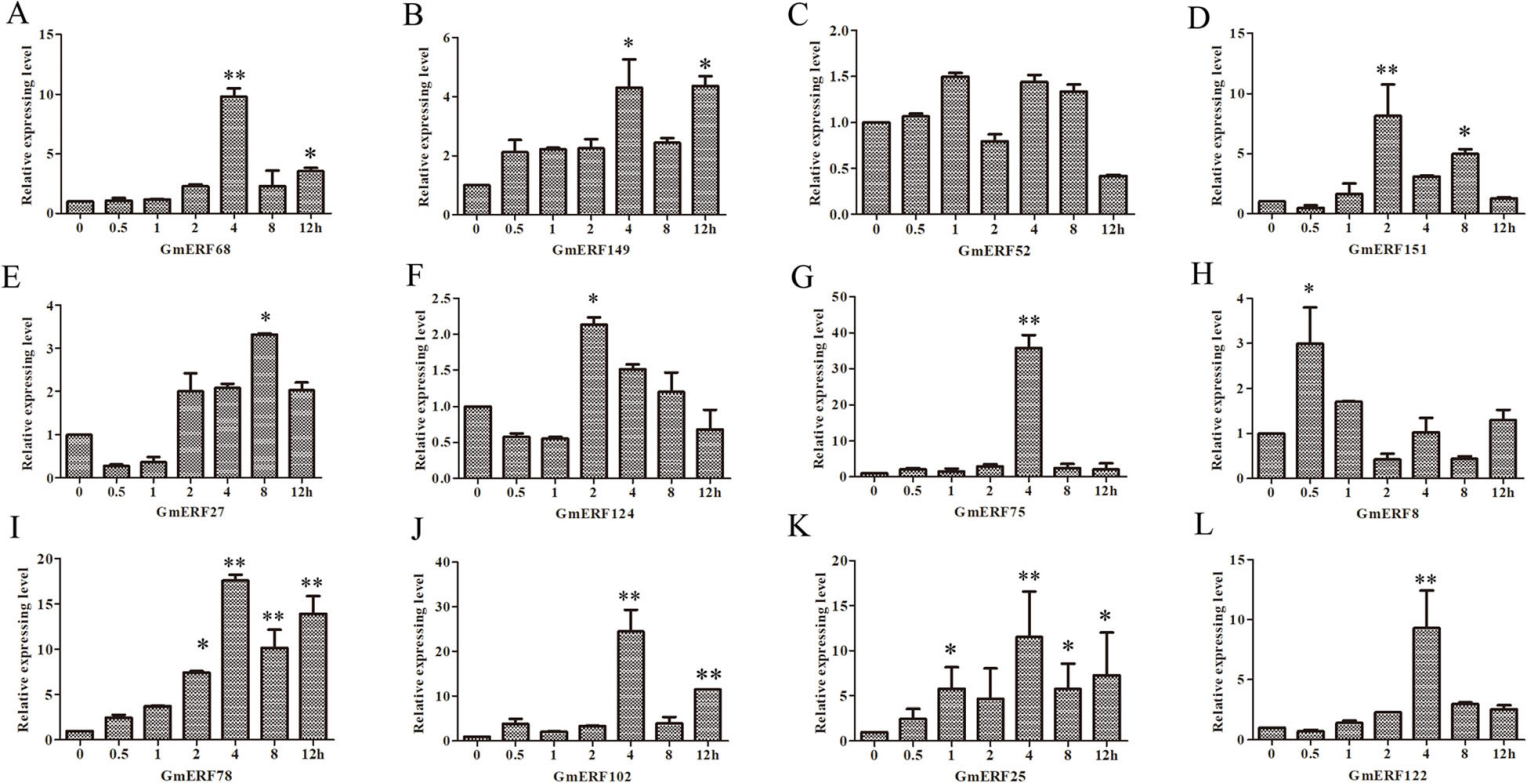
ABA-induced Group VII ERF genes expression. 14-day-old soybean seedlings were treated with ABA and collected the hypocotyl and roots 0, 0.5, 1, 2, 4, 8, 12 h after treatment for RNA extraction and qRT-PCR. Expression levels of the 12 Group VII ERFs in response to ABA treatment were revealed by qRT-PCR. The data was shown as the means±SD of three biological replicates

To investigate the expression levels of the 12 soybean ERFs after ABA treatment, quantitative real-time PCR (qRT-PCR) was conducted using cDNA obtained from hypocotyls and roots of ABA-treated soybean seedlings as a template. As shown in Fig. 3, almost all soybean ERFs were up-regulated to different extents in response to exogenous ABA treatment (Figs. 3a-l). Transcription level of *GmERF75* was the highest up-regulated and reached the highest level at 4 h after ABA treatment (Fig. 3g). Therefore, *GmERF75* was selected for further study.

To investigate the expression pattern of *GmERF75* in different soybean plant tissues, semi-quantitative PCR (semi-qPCR) was conducted. RNA was extracted from hypocotyls, roots, stems, and leaves of soybean seedlings. Parallel reactions amplifying Actin were performed to normalize the expression levels. This result showed that *GmERF75* is predominantly expressed in hypocotyls and roots, with less expression observed in leaves (Additional file 6: Figure S4).

### GmERF75 is localized in nucleus

The CDS of *GmERF75* was acquired that contained complete 903 bp open reading frame (ORF), which encodes a putative protein of 300 amino acids (Additional file 7: Figure S5). The GmERF75 protein contains a putative basic amino acid region (KPVKRQRK) that potentially act as a nuclear localization sequence (NLS), and acidic amino acid regions, EKETEVIEAEEEKNKVLELSEE and EEEEVVVEE, in the C-terminal region that may act as transcriptional activation domains (Additional file 7: Figure S5).

**Fig. 4:**
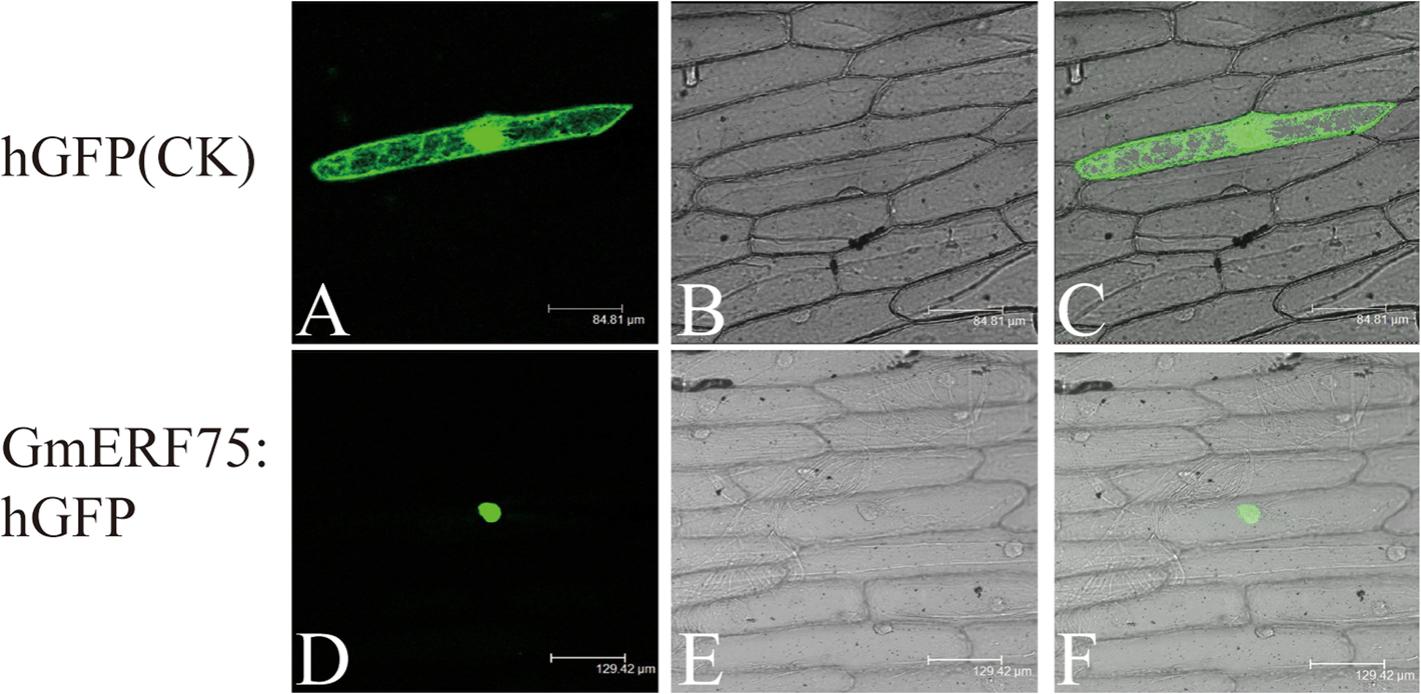
Subcellular localization of the GmERF75 protein. *GmERF75*-*hGFP* vector and control *hGFP* vector were bombarded into living onion epidermal cells. Localization of GmERF75 in onion epidermal cells was investigated using a confocal microscope (Leica). Photographs were taken in dark field to visualize green fluorescence (**a** and **d**) and in bright light to visualize cell morphology (**b** and **e**). Merged dark field and bright light images are shown in (**c** and **f**). Scale bars were shown in the bottom of each photo

To investigate whether the GmERF75 protein located in cell nucleus, the full-length ORF of *GmERF75* was amplificated and fused in frame with the *hGFP* gene under the control of the CaMV 35S promoter and then transferred into onion epidermal cells to observe fluorescence signal (Fig. 4). The result showed that GmERF75::hGFP fusion protein fluorescence was predominantly observed in the nucleus. GFP fluorescence of the control one was distributed throughout the cell. These results indicated that the GmERF75 fusion protein was targeted to nucleus.

### The *GmERF75* promoter region contains diverse stress-responsive elements

To further investigate the transcriptional regulation of *GmERF75*, 1809 bp promoter region of *GmERF75* upstream of the ATG start codon was isolated. Putative *cis*-acting elements in the promoter region were identified using the PLACE (http://www.dna.affrc.go.jp/PLACE/) and PlantCARE (http://bioinformatics.psb.ugent.be/webtools/plantcare) databases. Several distinct regulatory motifs homologous to *cis*-acting elements involved in responses to abiotic and biotic stresses and plant hormones were identified (Table 1).

**Table 1:** Analysis of putative *cis*-acting elements in the *GmERF75* promoter

*GmERF75*	*Cis*-acting elements	Core sequences	Functions
+	W-box	TTGACC	fungal elicitor responsive element
+	ACGT-element	ACGT	dehydration and dark-induced senescence
+	core of MYBST1	GGATA	ABA and stress responsive element
+	core of MYBST1	GGATA	ABA and stress responsive element
+	ABRE	ACGTG	ABA responsive element
+	DPBF binding site	ACACNNG	ABA responsive element
+	GARE	TAACAAR	gibberellin responsive element,
+	CAAT-box	CCAATT	common element in enhancer region
+	Box-4	ATTAAT	light responsive element
+	G-box	CACGAC	light responsive element
+	G-box	CACGTG/T	light responsive element
+	ACE	AAAACGTTTA	light responsive element
+	ACE	CTAACGTATT	light responsive element
+	TCA element	GAGAAGAATA	salicylic acid responsive element
+	TGA element	AACGAC	auxin responsive element
+	TC-rich repeat	ATTCTCTAAC	defense and stress responsive element
+	MYB binding site	WAACCA	ABA and stress responsive element
+	REα element	AACCAA	DNA binding activity is high in etiolated plants

Many abiotic and biotic stress-related *cis*-elements are distributed in the promoter region of *GmERF75*. There are eight hormone-responsive elements including five ABA relative elements (i.e., an AERB, two MYBST1 core binding site sequences, a DPBF binding site, and a MYB binding site), a gibberellic acid responsive element (GARE), a SA responsive element (TCA element), and an auxin responsive element (TGA element). Pathogen related elements (a W-box, and a TC-rich repeat) were also found in the promoter region. Interestingly, the REα element (AACCAA), which is highly bound in etiolated plants but lowly bound in green plants, was found in the *GmERF75* promoter region (Table 1). In addition, a series of light-responsive elements such as Box-4, G-box, ACE, and ACGT-element were also found in the *GmERF75* promoter region (Table 1). The presence of these *cis*-acting elements suggested that the expression level of *GmERF75* could be regulated by multiple stresses, which in turn indicated that *GmERF75* may participate in several signal transduction pathways.

### Changes in *GmERF75* expression in response to abiotic stresses and exogenous hormones

**Fig. 5:**
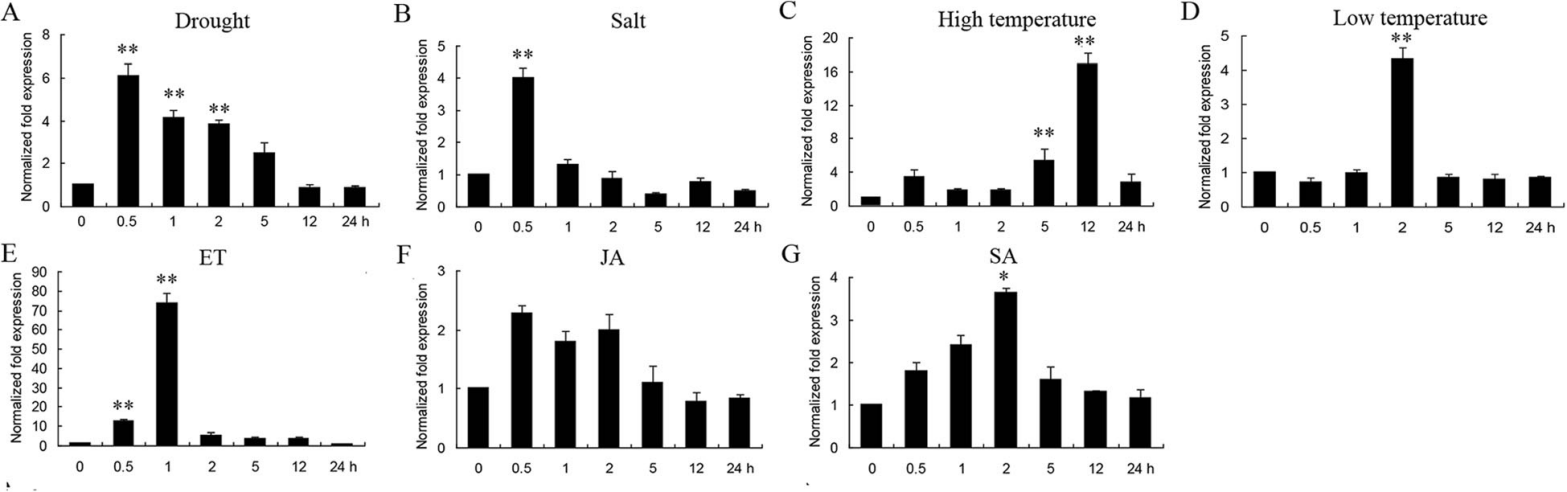
Changes in *GmERF75* expression in response to abiotic stress treatments and exogenous hormones. The kinetics of *GmERF75* mRNA accumulation were evaluated for hypocotyl and root of 14-day-old seedlings subjected to the abiotic stress treatments drought (**a**), NaCl (**b**), high temperature (**c**), and low temperature (**d**), or treated with the exogenous hormones ethylene (ET, **e**), jasmonate (JA, **f**), and salicylic acid (SA, **g**). The total RNA was extracted 0, 0.5, 1, 2, 5, 12, and 24 h after each treatment and used for qRT-PCR. The data was shown as the means±SD of three biological replicates

To investigate the expression level of *GmERF75* under abiotic stresses including drought, salt, and high/low temperature, and in the presence of exogenous hormones, qRT-PCR was conducted using total RNA extracted from hypocotyls and roots of soybean seedlings as a template. All of the treatments increased the expression level of *GmERF75*, particularly ET (about 75-fold increase). As shown in Fig. 5, *GmERF75* was rapidly induced by ET, exhibiting the highest increase in expression which has a 75-fold change within 1 h after ET treatment, and then expression gradually declined to normal level observed before treatment. Upon high temperature treatment, *GmERF75* expression peaked at 12 h (about 18-fold) and then declined to the initial level within 24 h (Fig. 5). Transcription of *GmERF75* was also up-regulated by drought (about 6-fold) and salt treatment (about 4-fold), and for both treatments expression levels were the highest at 0.5 h and declined to initial level within 24 h. Low temperature could increase GmERF75 transcription level by 4 times after 2 h of treatment. Expression levels also increased in response to exogenous SA. These results suggest that *GmERF75* may play a crucial role in numerous signal transduction pathways related to stress [30].

### *GmERF75* overexpression rescued two *Arabidopsis erf71* mutants hypocotyl elongation

To investigate the function of *GmERF75* in *Arabidopsis*, *AtERF71* was identified as a homologous gene of *GmERF75*, which share 55.47% identity compare to GmERF75. Two *Arabidopsis erf71* mutants (SALK_030459C, CS362782) were found to display shorter roots and hypocotyls compared with wild-type (WT) *Arabidopsis* [31] (Additional file 8: Figure S6). To assess whether *GmERF75* could rescue the phenotype of *erf71* mutants, *GmERF75* was introduced into the two mutants under the control of the CaMV 35S promoter, and transgenic *GmERF75::erf71* lines were obtained. T3 seeds of stable genetically inherited plants were used for further phenotypic analysis. Significant differences between WT and *erf71* mutants hypocotyl length were observed. The *erf71* mutants displayed shorter hypocotyls, while the *GmERF75::erf71* lines shared the similar phenotype with WT (Additional file 8: Figure S6). This result indicated that *GmERF75* could promote hypocotyl growth.

### *GmERF75* improved osmotic stress tolerance in transgenic *Arabidopsis* plants

**Fig. 6:**
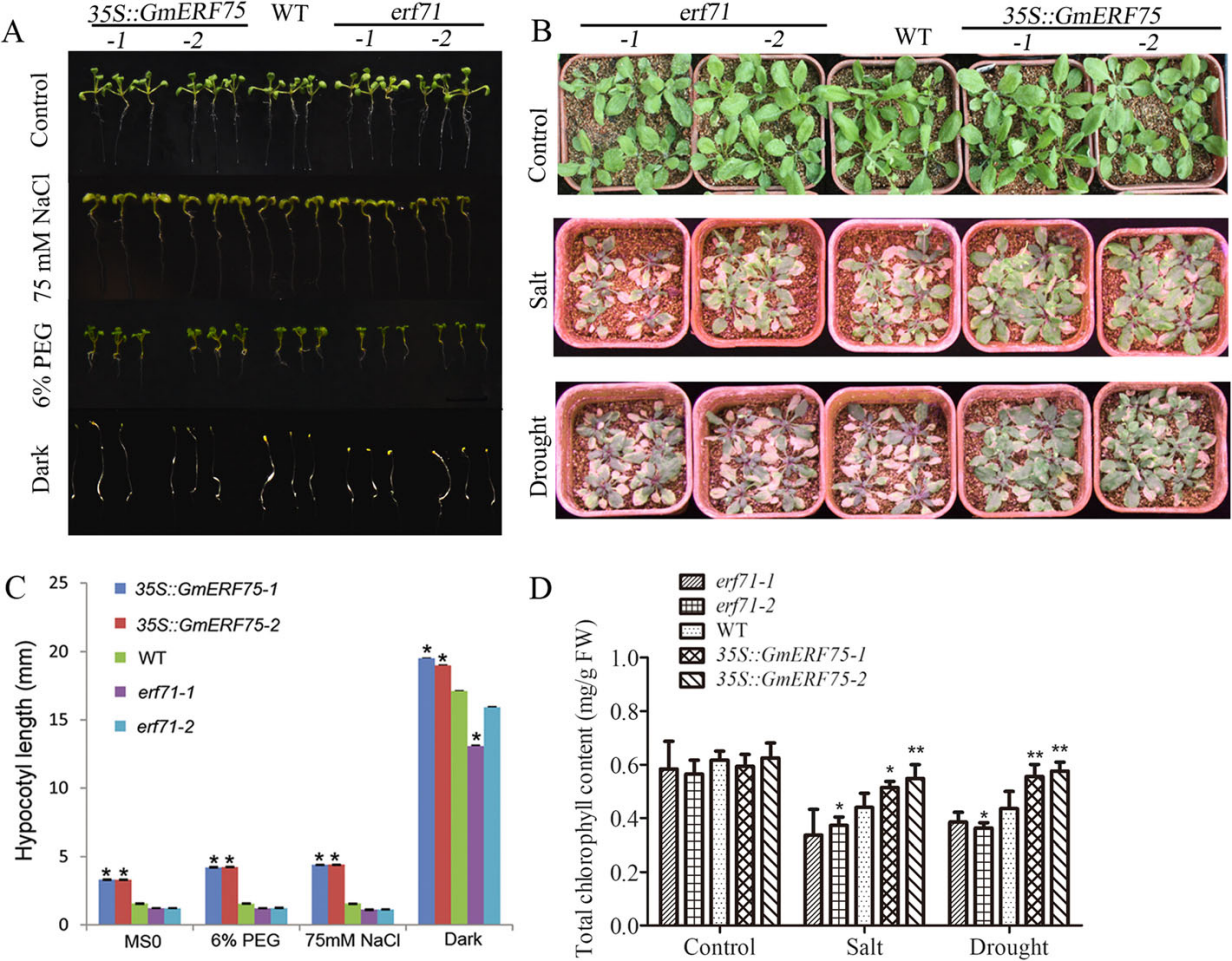
Overexpression of *GmERF75* in *Arabidopsis* enhanced tolerance of osmotic stress. **a** *GmERF75*-overexpressing lines had longer hypocotyls than the WT and the two *erf71* mutants. *Arabidopsis* seedlings of *GmERF75*-overexpressing, WT, and mutants grown on MS medium with/without 6% PEG, 75 mM NaCl, or dark condition, respectively. **b** Overexpression of *GmERF75* in *Arabidopsis* enhance the resistance to salt and drought. Three-week-old seedlings normally grown in soil were supplied with 250 mM NaCl for 2 weeks or were not watered for 1 week and then were re-watered for 1 week. **c** Hypocotyl length of 3*5S::GmERF75*, *erf71*, and WT. Fifteen to twenty-five individuals in each treatment were used to count the hypocotyl length. The data was shown as the means ± SD of three biological replicates. **d** Total chlorophyll content of the mutants, WT (Col-0), and GmERF75 transgenic lines under drought and salt treatment. Three-week-old seedlings of mutants, WT (Col-0), and transgenic lines were supplemented with 250 mM NaCl or un-watered for 1 week, and recovered for 1 week. 0.1 g leaves of each line were collected and used to measure chlorophyll content. The data was shown as the means±SD of five repetitions each lines. Asterisks indicate significant differences from WT at *P < 0.05 and **P < 0.01 determined by Student’s *t* test

The *GmERF75* gene was strongly induced by various abiotic stresses (Fig. 5). To evaluate the contribution of the *GmERF75* gene to abiotic stress tolerance, two *GmERF75*-overexpressing *Arabidopsis* lines were grown under PEG, NaCl, and dark conditions. The *GmERF75*-overexpressing lines displayed longer hypocotyls under different abiotic stresses than WT *Arabidopsis* plants (Fig. 6a). The largest differences in hypocotyl length between the *35S::GmERF75* lines and WT were observed after 5 days of treatment with 75 mM salt and 6% PEG (Fig. 6c).

To test the tolerance to salt and drought in late stage of *Arabidopsis*, three-week-old seedlings were treated with 250 mM NaCl for 2 weeks or not watered for 1 week and then re-watered (Fig. 6b). The chlorophyll content of each line were recorded (Fig. 6d). The result showed that the chlorophyll content of transgenic plants under salt treatment was increased by 20.11 and 39.66% compared to WT and the mutants, respectively. For drought treatment, the chlorophyll content of transgenic plants was increased by 29.70% compared to WT, Taken together, these results suggest that *GmERF75* has a role in improving tolerance to osmotic stress in *Arabidopsis*.

### *GmERF75* improved tolerance to salt stress and exogenous ABA in transgenic soybean hairy roots

**Fig. 7:**
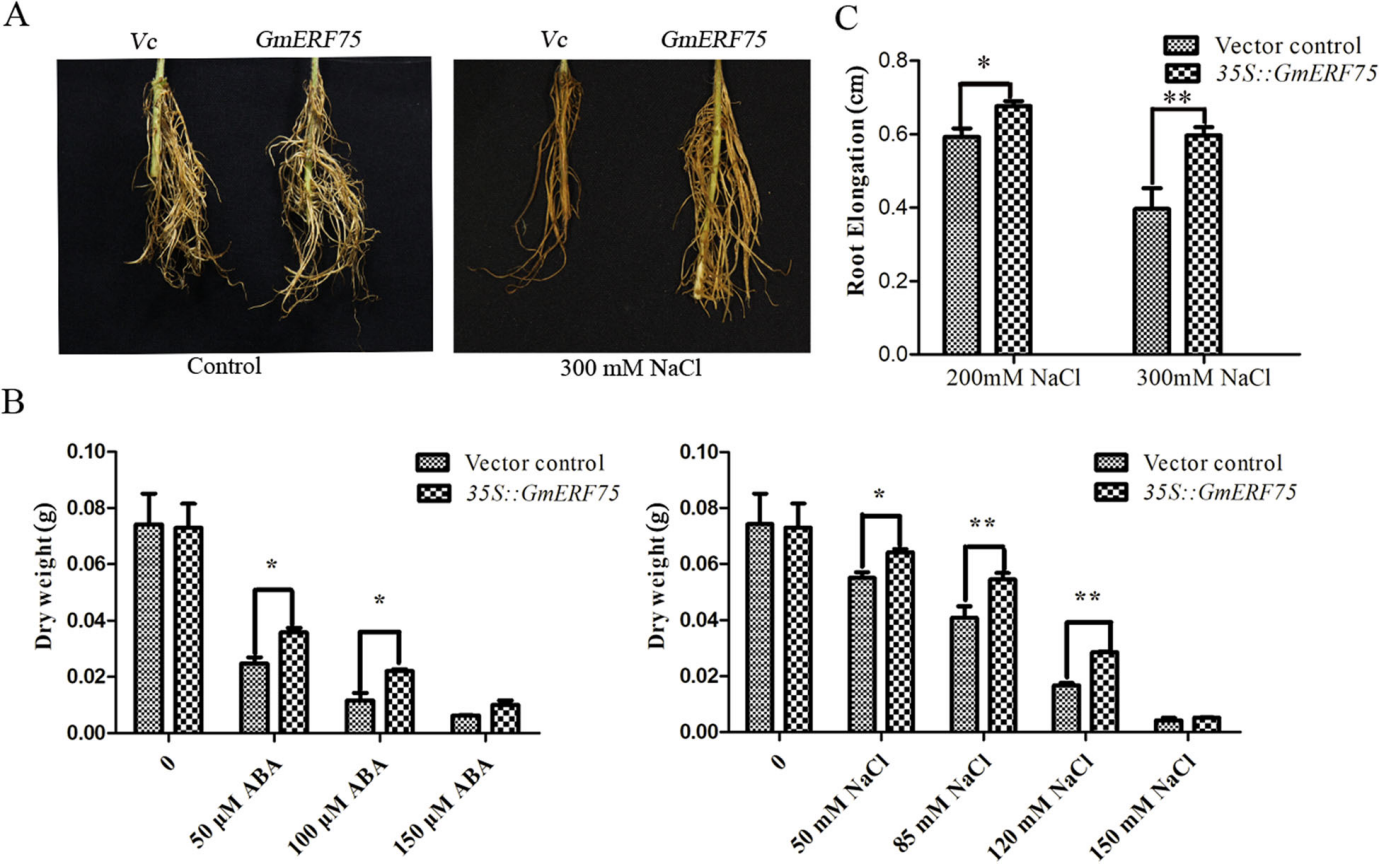
*GmERF75* improved salt tolerance in soybean hairy roots. **a** Hairy roots separately transformed with the binary vector *p*GFPGUS*Plus*-*GmERF75* and the vector control *p*GFPGUS*Plus* (Vc) using *A. rhizogenes* K599 were cultured on 1/2 MS medium that was supplemented with or without 300 mM NaCl for 2 weeks. Images were taken at the end of the stress treatment. **b** Root dry weights of *p*GFPGUS*Plus*-*GmERF75* and Vc hairy roots grown in the presence of different concentrations of NaCl or ABA. Data are shown as means ± SD of three independent replicates for each treatment. **c** Lengths of *p*GFPGUS*Plus*-*GmERF75* and Vc hairy roots after different concentration of NaCl. Asterisks indicate significant differences from the vector control at *P < 0.05 and **P < 0.01 determined by Student’s *t* test

To further investigate the function of *GmERF75* in stress tolerance in soybean, a *p*GFPGUS*Plus* vector designed to express *p*GFPGUS*Plus*-*GmERF75* was constructed and then transformed into Cucumopine-type *Agrobacterium rhizogene* strain K599, which was injected into *Superroot* of *Lotus corniculatus*. The positive transgenic hairy roots cultured on 1/2 Murashige and Skoog (MS) medium containing PEG, NaCl, or ABA, which were verified via GFP fluorescence. Transgenic hairy roots were much longer than vector control hairy roots under NaCl treatment in seedling stage (Fig. 7a). The higher dry weights of transgenic hairy roots also supported this conclusion (Fig. 7b). As shown in Fig. 7b, transgenic hairy roots transformed with *p*GFPGUS*Plus*-*GmERF75* exhibited more growth than those transformed with the empty vector control under different concentrations of NaCl and ABA. Extremely significant differences between the transgenic and control hairy roots were observed under 85 and 120 mM NaCl treatment, and significant differences were also observed under 50 and 100 μM ABA. However, there was no obvious difference between transgenic and vector control hairy roots under the PEG condition (data not shown). These results suggested that *GmERF75* could improve salinity and exogenous ABA tolerance in soybean.

## Discussion

Transcription factors function as either activators or repressors that up-regulate or down-regulate, respectively, a whole array of target genes, overexpression of which can modulate stress tolerance in plants [32]. Numerous transcription factors has been reported involving in defense against multiple abiotic and biotic stimulus in plants, such as WRKY [33, 34], MYB [35], NAC [36], and ERF [30, 37]. Therefore, the identification and functional analysis of new transcription factor genes is of great importance for understanding the molecular mechanisms of stress tolerance in plants, which may aid efforts to improve crop productivity. ERF transcription factors have been shown to be involved in the response to environmental stresses [5]. In this study, a comprehensive set of 160 soybean ERFs was identified and characterized. To better understand ERF-mediated stress responses, a highly ABA-induced soybean ERF, *GmERF75*, was isolated and its involvement in stress signal transduction pathways was investigated.

### *GmERF75* may integrate the SA and ET/JA pathways

The signal transduction pathways under abiotic stress were extremely complicated and complex in higher plants [38]. Hormones signaling transduction pathways were associated with different environmental stresses when plants resist various stresses, such as drought, salt, cold. It has been verified that there is an antagonistic effect between SA and JA pathways and between the JA/ET and ABA pathways which could precisely regulated the stress-related gene expression [39–41]. Accordingly, the expression levels of some plant defense genes are impacted via multiple signaling pathways during defense responses [42].

It is known that certain ERF transcription factors are targets of different signaling pathways [5]. For example, *ERF1* can be activated rapidly by ET or JA or synergistically activated by both [43, 44]. *AtERF4*, which acts as a transcriptional repressor, can be induced by both ET and JA [13, 45]. Meanwhile, the SA signal transduction pathway can act antagonistically with the ET/JA pathway [46, 47]. However, in this study, the *GmERF75* gene could be induced by exogenous SA, JA, and ET, which indicates that the transcription of *GmERF75* can be activated by both the SA and JA/ET pathways (Fig. 5) [48]. These results indicate that *GmERF75* may integrate signals from the SA and ET/JA pathways but does not contribute to the antagonistic interplay between them during the soybean seedling stage.

### The role of *GmERF75* in enhancing hypocotyl length

Hypocotyl elongation is regulated by a combination of extrinsic and intrinsic signals, including light and plant hormones [49–51]. Plants have evolved a complicated network of photoreceptors and numerous downstream signaling factors that enable them to respond and adapt to the ambient light environment [52]. vonArnim et al. found that *Arabidopsis* seedlings grown under light displayed short hypocotyls and open cotyledons with functional chloroplasts via photomorphogenesis, while dark-grown plants exhibit long hypocotyls and closed cotyledons and develop etioplasts via a process termed etiolation or skotomorphogenesis [53]. It was reported that light is closely related to hypocotyl cell elongation [54, 55], and that photoreceptors can modulate downstream transcription factors, such as ELONGATED HYPOCOTYL5 (HY5) [56]. HY5 can indirectly affect the transduction of many hormone signal transduction pathways, such as ABA, ET, and JA [57]. In this study, *GmERF75* was mainly expressed in hypocotyls (Additional file 6: Figure S4) and could be induced by exogenous ABA, ET, and JA (Fig. 5), which suggested *GmERF75* functions downstream of these hormone signaling pathways. The *erf71* mutants displayed shorter hypocotyls, while the hypocotyls of *GmERF75::erf71* lines were not significantly different in length to WT hypocotyls (Additional file 7: Figure S5). These results implied that *GmERF75* may participate in the light-photoreceptor-HY5-ABA/ET/JA signal transduction pathway to modulate hypocotyl growth. In addition, promoter analysis showed there are six light-responsive *cis*-elements in the promoter region of *GmERF75*, which suggested that this gene may be directly regulated by light. Taken together, these results suggested that GmERF75 may regulate hypocotyls elongation through light-related signaling pathways.

### *GmERF75* may be an essential factor in diverse abiotic signaling pathways

It is well known that there are complex connections among various hormones and stress signaling pathways in plants, and a single gene may play roles in many different signaling pathways at same time. Overexpression of *JcDREB2*, a physic nut AP2/ERF gene, in rice can suppress the expression of some gibberellic acid biosynthetic genes and induce salt tolerance-related genes to regulate salt stress response [58]. *AhDREB1* is an important member of the AP2/ERF family in peanut. *Arabidopsis* plants overexpressing *AhDREB1* had higher ABA sensitivity compared with WT and the expression levels of downstream drought stress-related genes were altered, which demonstrated that overexpression of *AhDREB1* could improve tolerance to drought by affecting the ABA-dependent pathway [59]. Similarly, transgenic tobacco plants expressing *GmERF9* had enhanced tolerance to drought and cold stresses and increased expression levels of PR genes such as PR1 and PR2 [60]. In this study, both transgenic *Arabidopsis* plants and soybean hairy roots expressing *GmERF75* showed high salt stress tolerance and lower ABA sensitivity. These results suggested that *GmERF75* may be involved in salt- and ABA-related signaling pathways. Based on these findings, we conclude that *GmERF75* encodes a transcription factor that is likely to be an important determinant of osmotic stress signal transduction pathways in *Arabidopsis* and soybean.

## Conclusion

GmERF75, protein localized in the nucleus, is responsive to multiple abiotic stresses and exogenous hormones. Two independent *Arabidopsis* mutations of *AtERF71*, a gene homologous to *GmERF75*, displayed shorter hypocotyls, and overexpression of *GmERF75* in these mutants could rescue the short hypocotyl phenotypes. *GmERF75*-overexpressing *Arabidopsis* lines showed higher chlorophyll content under drought and salt stress. Overexpressing *GmERF75* in soybean hairy roots improved root growth under exogenous ABA and salt stress. GmERF75 is an important plant transcription factor that plays a critical role in enhancing osmotic tolerance in both *Arabidopsis* and soybean.

## Methods

### Database searches and the chromosomal distribution of ERF genes in the soybean genome

The whole genome sequence and repeat information for soybean were obtained from the JGI Glyma1.0 annotation (http://www.phytozome.net/index.php) [61]. The gene chip data for soybean were obtained from SoyBase (http://www.soybase.org/) [62]. The chromosomal distribution was determined using the chromosome locus information from Phytozome. The MapInspect program was used to draw the chromosomal distribution map.

### Alignment and phylogenetic analysis

We used the Pfam [24] (http://pfam.sanger.ac.uk/) and SMART databases [25] (http://smart.embl-heidelberg.de/) as references for the identification of 160 non-redundant soybean ERFs (Additional file 1: Table S1). Amino acid sequence alignments were performed using ClustalX and were manually corrected. Neighbor-joining method was used to construct the phylogenetic tree of soybean ERFs by MEGA 5.1 [26].

### Expression profiles and gene structure analysis

Expression analysis was conducted using soybean GeneChip expression data for different tissues and developmental stages. The genomic DNA sequences and corresponding coding sequences of the 12 soybean ERF genes were submitted to the Gene Structure Display Server (GSDS) website (http://gsds.cbi.pku.edu.cn/) to visualize the gene structures [63]. The conserved motifs were analyzed using multiple EM for motif elicitation (MEME) software. The sequences were aligned using DNAMAN software.

### Protein domain and homology modeling

The amino acid sequences of the 12 Group VII ERF genes were submitted to the Protein Fold Recognition Server (PHYRE2) (http://www.sbg.bio.ic.ac.uk/phyre2/html/page.cgi?id=index) for structural homology modeling. DOG 2.0 was used to draw the protein domains.

### Plant materials and stress treatments

Soybean seedlings (*Glycine max* cv. Tiefeng 8) grown in soil at 25 °C for 14 days were subjected to various abiotic stress and exogenous hormone treatments. To investigate the effects of exogenous ABA on ERF transcript family, the soybean seedlings were incubated in 100 μM ABA for 0, 0.5, 1, 2, 4, 8, or 12 h [64]. To investigate the effects of abiotic stresses on ERF transcript family, seedlings were subjected to stress for 0, 0.5, 1, 2, 5, 12, or 24 h. For rapid induction of drought stress, seedlings were exposed to air on filter paper [65]. For cold stress, seedlings were placed in a 4 °C chamber [66]. For high-temperature treatment, seedlings were placed in a 42 °C oven, and for salt stress, seedlings were incubated in 200 mM NaCl [35]. To investigate the effects of the exogenous hormones SA and JA on physiological and molecular responses, seedlings were incubated in 50 μM SA and 50 μM JA, respectively for 0, 0.5, 1, 2, 5, 12, or 24 h. To evaluate the response to ET, seedlings were placed in a sealed plastic box with a concentration of 200 μl 1^− 1^ by injection of ethylene for 0, 0.5, 1, 2, 5, 12, or 24 h [20, 67, 68]. For each treatment, 42 individuals were distributed to three groups for three sample replicates. Hypocotyls and roots of two individuals were collected as a sample at each time point, frozen immediately in liquid nitrogen, and stored at − 80 °C for RNA extraction. There are three repetitions at each time point of each treatment.

### RNA extraction, semi-qPCR, and qRT-PCR

Trizol reagent was used to extract total RNA of the hypocotyls and roots according to the protocol (TIANGEN, China). After treated by DNase I, total RNA was used to synthesize cDNA using PrimeScript First-Strand cDNA Synthesis Kit (TaKaRa, Japan). Semi-qPCR was conducted to investigate the expression pattern of *GmERF75* in different soybean plant tissues. RNA was extracted from hypocotyls, roots, stems, and leaves of soybean seedlings. Parallel reactions amplifying actin were performed to normalize the expression levels. qRT-PCR was used to analyze the expression patterns of several soybean ERF genes in response to various abiotic stresses and exogenous hormones. qRT-PCR analysis of soybean ERFs was performed using the SYBR Premix Ex Taq™ kit (TaKaRa, Japan) according to the manufacturer’s protocol. The expression patterns were analyzed using ABI Prism 7500 sequence detection system (ThermoFisher Scientific, USA) as previously described [69, 70]. The soybean ERF gene primers for qRT-PCR were designed to anneal to regions outside the conserved AP2/ERF domain using Primer Premier 5.0 software, and soybean Actin (U60506) [71] was used as an internal control for normalizing the amount of template cDNA. The primers used for qRT-PCR are listed in Additional file 9: Table S3.

### Cloning of *GmERF75*

The full-length ORF of *GmERF75* was amplified from soybean cDNA using the primers 5′-ATGGCGAACGCAGCTGAAGTTT-3′ and 5′-TCACACCGCCACGAGCG-3′. The PCR product was cloned into the *p*EASY-T1 vector (TransGen, China).

### Subcellular localization assay

To investigate the biological activity of the putative NLSs, the full-length cDNA sequence of *GmERF75* was fused to the N-terminus of the *humanized green fluorescent protein* (*hGFP*) gene under the control of the double Cauliflower Mosaic Virus (2 × CaMV) 35S promoter. The recombinant plasmid and control plasmid (*hGFP* vector) were bombarded into living onion epidermal cells. Visualization of *hGFP* expression in the onion epidermal cells was performed as described previously [20, 33].

### Generation of transgenic *Arabidopsis* and stress treatments

The coding sequence of *GmERF75* was amplified using the primers 5′-TGATTACGCCAAGCTTATGGCGAACGCAGCTGAAGTTT-3′ and 5′-CCGGGGATCCTCTAGACACCGCCACGAGCG-3′ and cloned into pBI121 under the control of the CaMV 35S promoter to generate the *35S::GmERF75* construct. The construct was confirmed by sequencing and then transformed into WT *Arabidopsis* plants (Col-0) using the vacuum infiltration method [72, 73]. The transgenic Arabidopsis seeds were screened and T3 seeds of two transgenic lines were used for further phenotypic analysis.

For phenotype analysis, *GmERF75* overexpression, *erf71* mutant, and WT *Arabidopsis* seedlings at the two-leaf stage were transferred to MS medium containing 6% PEG, 75 mM NaCl, or placed in dark. For each treatment, fifteen to twenty-five individuals of each line were used to count the hypocotyl length. Three independent biological replicates were performed for each treatment.

To test the resistance of salt and drought in late stage, 72 *Arabidopsis* seedlings of *GmERF75* overexpression, *erf71* mutant, and WT, respectively, were transferred into soil for normally growth after germinating on the MS medium. For each line, all the seedlings were divided into 12 pots, each of which planted 6 seedlings per pot. Three-week-old seedlings were supplied with 250 mM NaCl 2 weeks for salt treatment. Three-week-old seedlings normally grown in soil were not watered for drought treatment. A week later, different phenotypes were observed. The *Arabidopsis* plants were re-watered and recovered for 1 week, and the leaves of each line were collected. Plants normally watered were used as a control. Three independent replicates were performed for each treatment. In order to quantify the phenotype of *Arabidopsis* response to salt and drought, the chlorophyll content of each line were determined according to the protocol (Cominbio, China). Take 0.1 g of *Arabidopsis* leaves of each line and wash them with distilled water. Add 1 mL of 80% acetone, mix well and leaching overnight until the leaves are completely white. Add 80% acetone to 1 mL cuvette and zero the cuvette. The absorbance values of the samples at 663 nm and 645 nm were measured and recorded as A_663_ and A_645_. Total chlorophyll content (mg/g FW) = (20.21*A_645_ + 8.02*A_663_)*1 mL/0.05 g/1000.

### Soybean hairy root induction and stress treatments

Seedling growth, rooting, hairy root induction, and hairy root transformation were performed as described by Chen et al. [74, 75]. Chlorine gas-sterilized soybean seeds were germinated in B5 medium. The cotyledons of 4-day-old seedlings as explant were harvested and wounded with a scalpel with K599 carrying the *p*GFPGUS*Plus*-*GmERF75* binary vector for 5 days growth, which was used to transform *Superroot*-derived *L. corniculatus* plants for about 11 days to observe the hairy roots. The positive transgenic hairy roots were verified via fluorescence GFP. Then a total of 256 GFP-positive (GFP^+^) hairy roots were cultured on 1/2 MS medium that was supplemented with 50, 85, 120 or 150 mM NaCl, or 50, 100, or 150 μM ABA and incubated at 24 °C under a 16/8 h light/dark cycle for 2 weeks. After 24 h incubation at 105 °C, the dry weight increment (30 roots per unit) was calculated and recorded.

The cotyledonary leaf nodes of 45 soybean seedlings (*Glycine max* cv. Tiefeng 8) grown in vermiculite at 25 °C for 7 days were infected by *p*GFPGUS*Plus*-*GmERF75* and vector control. After growing in the soil for about 20 days, the hairy roots will sprout out. The transgenic hairy roots and the control were supplied with different concentrations of NaCl treatment for 1 week then the root elongation was measured. Three independent replicates were performed for each treatment.

### Statistical analysis

For experiments with single time point, three biological repetitions were performed. For experiment with multiple time points, three independent biological repetitions and three technical repetitions were performed. The data was shown as the means ± SD of all of the replicates. Asterisks indicate significant difference or extremely significant difference from the control at *P < 0.05 or **P < 0.01, which was determined by Student’s *t* test.

## Supplementary information

**Additional file 1: Table S1.** Genetic information for soybean ERFs.

**Additional file 2: Figure S1.** Analysis of soybean ERF expression in different organs and developmental stages. Normalized expression data for the soybean ERFs were collected from SoyBase (http://www.soybase.org/) (Additional file 3: Table S2). The expression levels (vertical coordinates) are reported in transcripts per million (TPM). The different tissues and developmental stages are shown under the horizontal ordinate. The different colors indicate the expression levels of soybean ERFs.

**Additional file 3: Table S2.** Expression data during different organs and development periods of soybean ERFs.

**Additional file 4: Figure S2.** Protein domains in the 12 soybean ERF proteins. DOG 2.0 was used to draw the domains in each protein. The conserved AP2/ERF domain is indicated by blue boxes.

**Additional file 5: Figure S3.** Intron-exon structures of the 12 soybean ERF genes. The diagrams of intron-exon structure were generated using the GSDS online tool. The exons, introns, and untranslated regions (UTRs) are indicated by yellow boxes, black lines, and blue boxes, respectively.

**Additional file 6: Figure S4.** *GmERF75* expression in specific tissues of soybean plants under normal growth conditions. RNA was extracted from hypocotyls, roots, stems, and leaves of soybean seedlings. Parallel reactions amplifying Actin were performed to normalize the expression levels.

**Additional file 7: Figure S5.** Nucleotide and deduced amino acid sequences of the *GmERF75* gene. Untranslated regions (UTRs) and intron sequences are indicated by lowercase letters. The deduced amino acid sequence is shown below the DNA sequence. The AP2/ERF domain is underlined. Basic amino acid regions that potentially act as nuclear localization signals are outlined by boxes, and an acidic amino acid region that may act as a transcriptional activation domain is shown in bold italics. A potential *N*-linked glycosylation site is indicated by a dotted line.

**Additional file 8: Figure S6.** *GmERF75* rescued the short hypocotyl length phenotype of two *erf71* mutants. (A) The *erf71* mutants displayed shorter hypocotyls than the WT. (B) Overexpression of *GmERF75* in the mutants partially rescued the short hypocotyl length phenotype. The histogram on the right shows the distribution of hypocotyl lengths for at least 30 seedlings.

**Additional file 9: Table S3.** Primers used for qRT-PCR of soybean ERFs in Group VII.

## Data Availability

The datasets using for the present study are available in the JGI Glyma1.0 repository, https://phytozome.jgi.doe.gov/pz/portal.html.

## Abbreviations

ABA: Abscisic acid
ACC: 1-aminocyclopropane-l-carboxylic acid
AD: Activation domain
AP2: APETALA2
CaMV: Cauliflower mosaic virus
DPBF: DO promoter-binding factors
DRE/CRT: Dehydration-responsive element/C-repeat
EREBP: Ethylene-responsive element binding protein
ERF: Ethylene-responsive factor
ET: Ethylene
GFP: Green fluorescent protein
GUS: β-glucuronidase
JA: Jasmonic acid
qRT-PCR: quantitative real-time PCR
RT-PCR: Reverse transcription PCR
SA: Salicylic acid
WT: Wild type
