# Supplementary figures and images for: The ABA-induced soybean ERF transcription factor gene GmERF75 plays a role in enhancing osmotic stress tolerance in Arabidopsis and soybean
Source: BMC Plant Biol. 2019 Nov 20;19:506. doi: 10.1186/s12870-019-2066-6 (PMC6865046; doi:10.1186/s12870-019-2066-6)

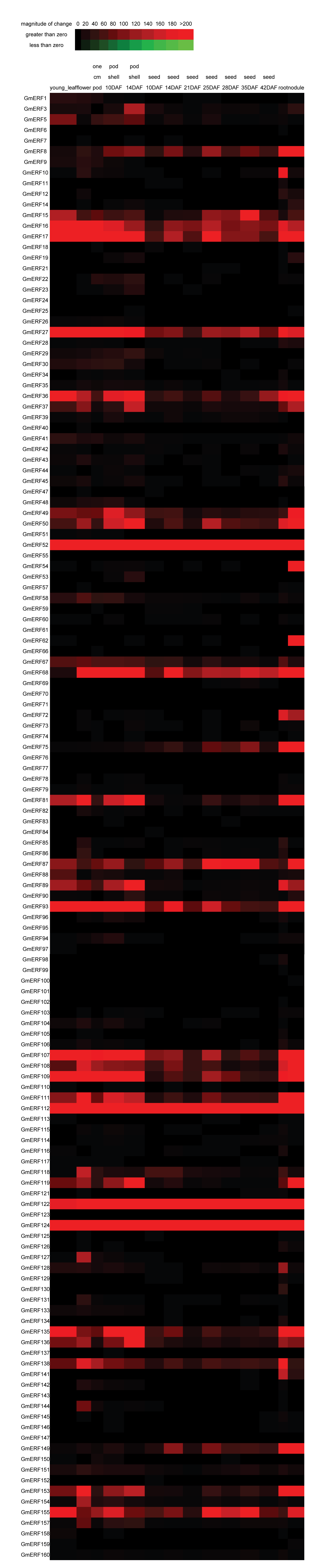

Supplement: Supplementary file 2 — Additional file 2: Figure S1. Analysis of soybean ERF expression in different organs and developmental stages. Normalized expression data for the soybean ERFs were collected from SoyBase (http://www.soybase.org/) (Additional file 3: Table S2). The expression levels (vertical coordinates) are reported in transcripts per million (TPM). The different tissues and developmental stages are shown under the horizontal ordinate. The different colors indicate the expression levels of soybean ERFs. [file 12870_2019_2066_MOESM2_ESM.tif]

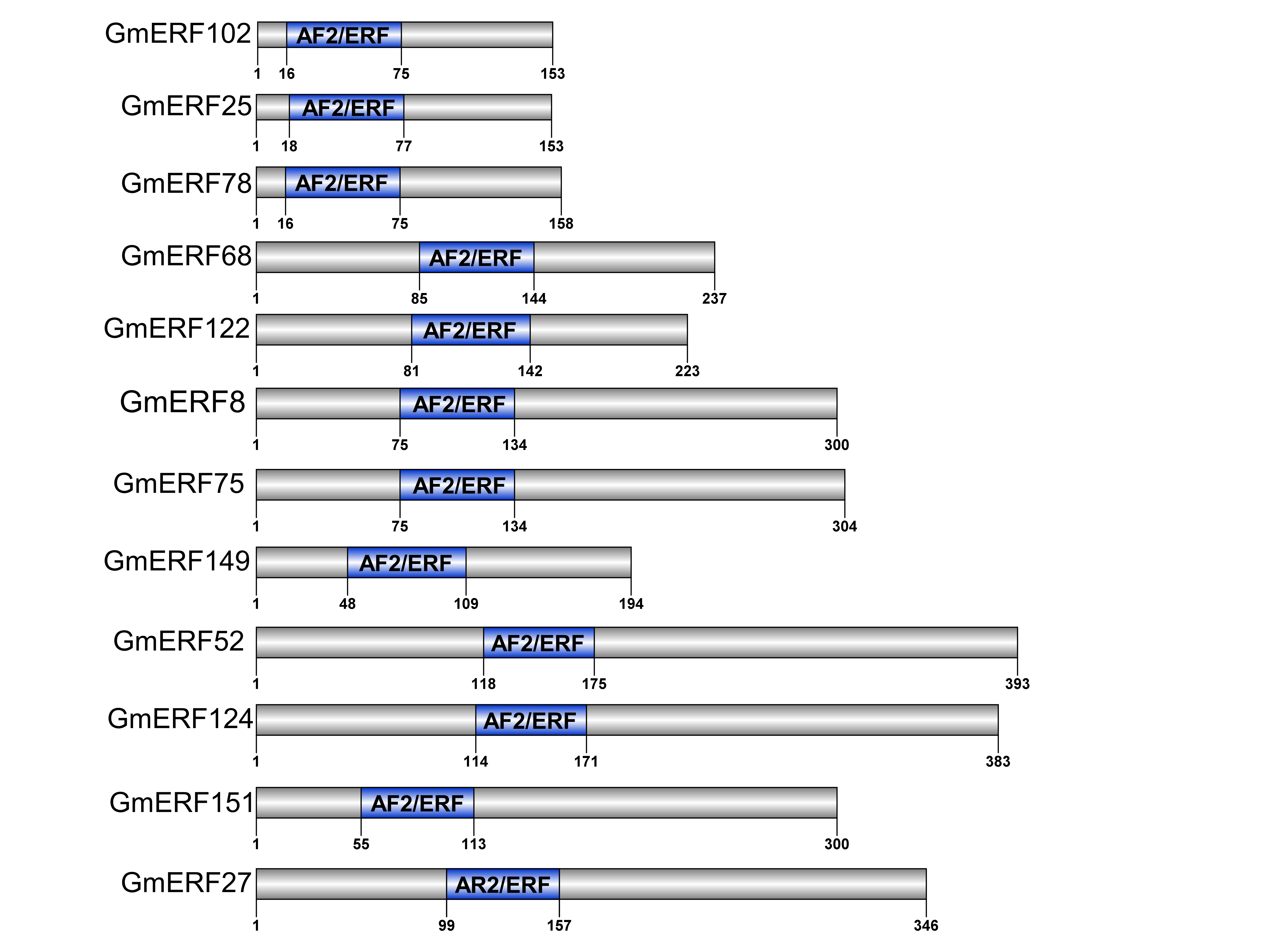

Supplement: Supplementary file 4 — Additional file 4: Figure S2. Protein domains in the 12 soybean ERF proteins. DOG 2.0 was used to draw the domains in each protein. The conserved AP2/ERF domain is indicated by blue boxes. [file 12870_2019_2066_MOESM4_ESM.tif]

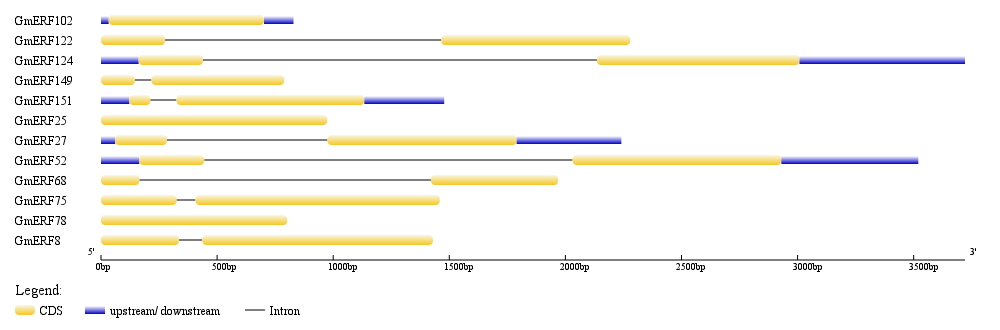

Supplement: Supplementary file 5 — Additional file 5: Figure S3. Intron-exon structures of the 12 soybean ERF genes. The diagrams of intron-exon structure were generated using the GSDS online tool. The exons, introns, and untranslated regions (UTRs) are indicated by yellow boxes, black lines, and blue boxes, respectively. [file 12870_2019_2066_MOESM5_ESM.png]

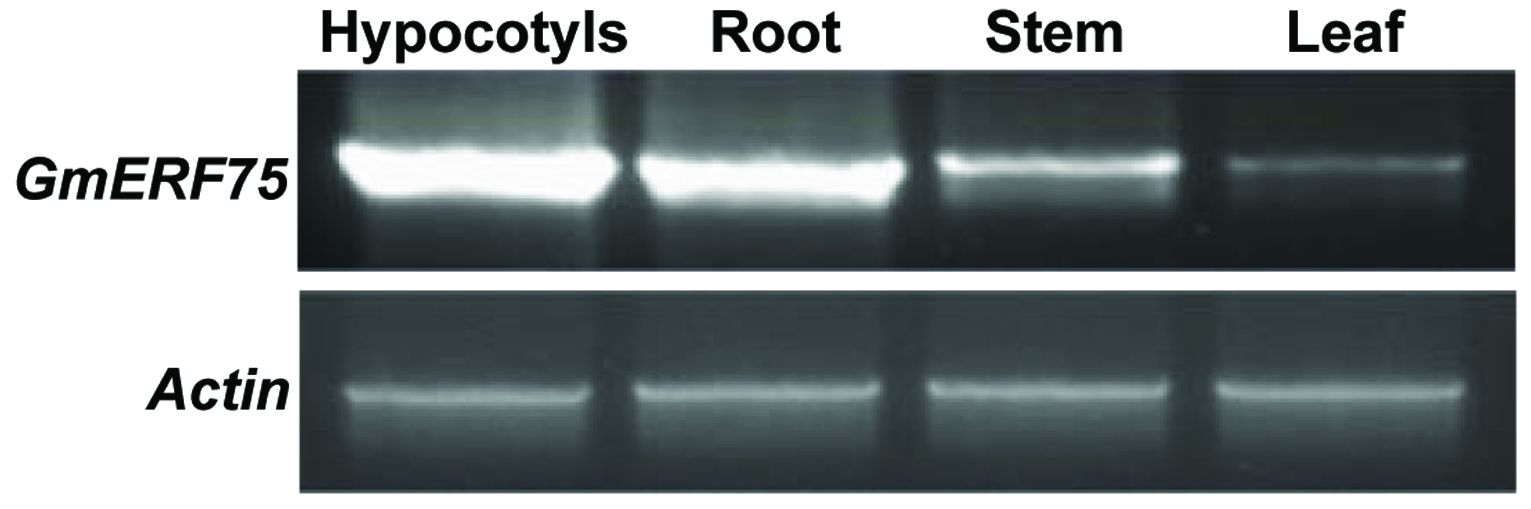

Supplement: Supplementary file 6 — Additional file 6: Figure S4. GmERF75 expression in specific tissues of soybean plants under normal growth conditions. RNA was extracted from hypocotyls, roots, stems, and leaves of soybean seedlings. Parallel reactions amplifying Actin were performed to normalize the expression levels. [file 12870_2019_2066_MOESM6_ESM.tif]

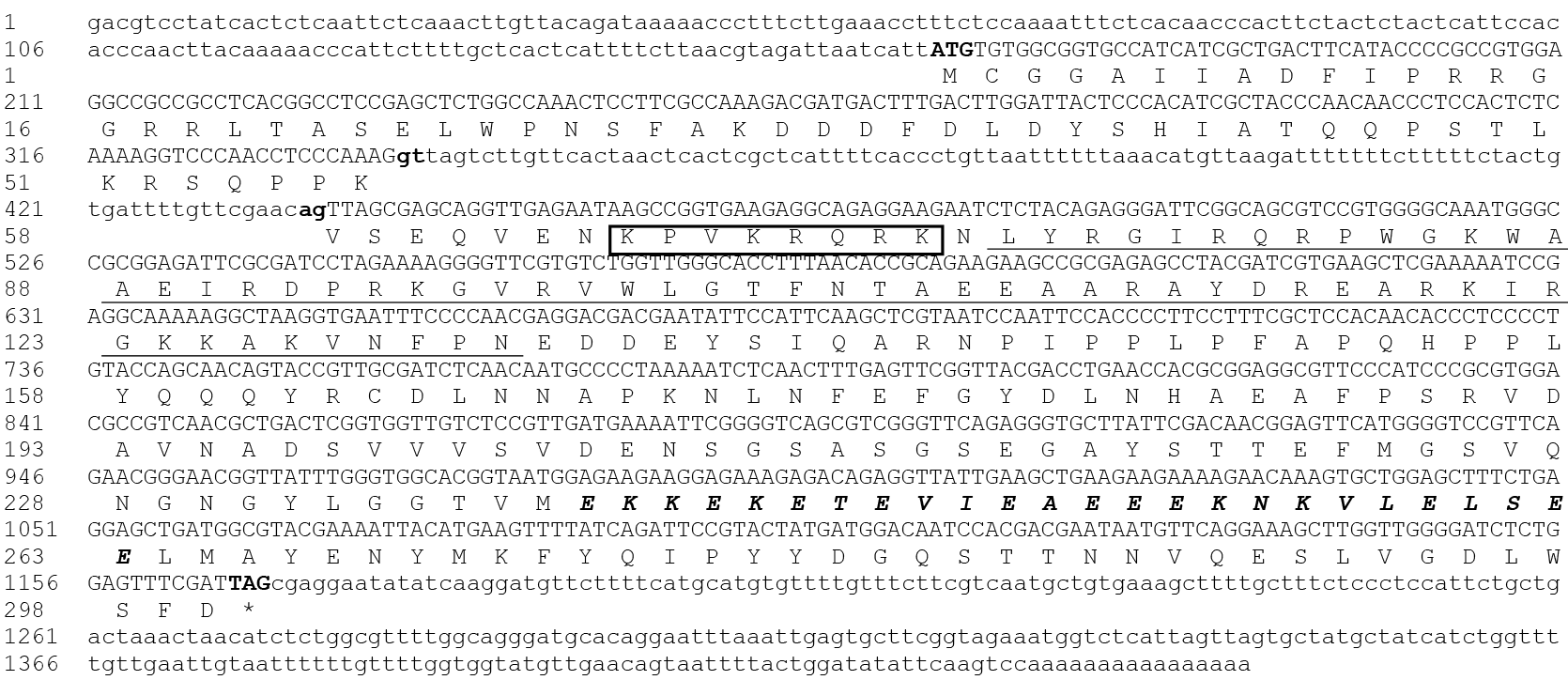

Supplement: Supplementary file 7 — Additional file 7: Figure S5. Nucleotide and deduced amino acid sequences of the GmERF75 gene. Untranslated regions (UTRs) and intron sequences are indicated by lowercase letters. The deduced amino acid sequence is shown below the DNA sequence. The AP2/ERF domain is underlined. Basic amino acid regions that potentially act as nuclear localization signals are outlined by boxes, and an acidic amino acid region that may act as a transcriptional activation domain is shown in bold italics. A potential N-linked glycosylation site is indicated by a dotted line. [file 12870_2019_2066_MOESM7_ESM.tif]

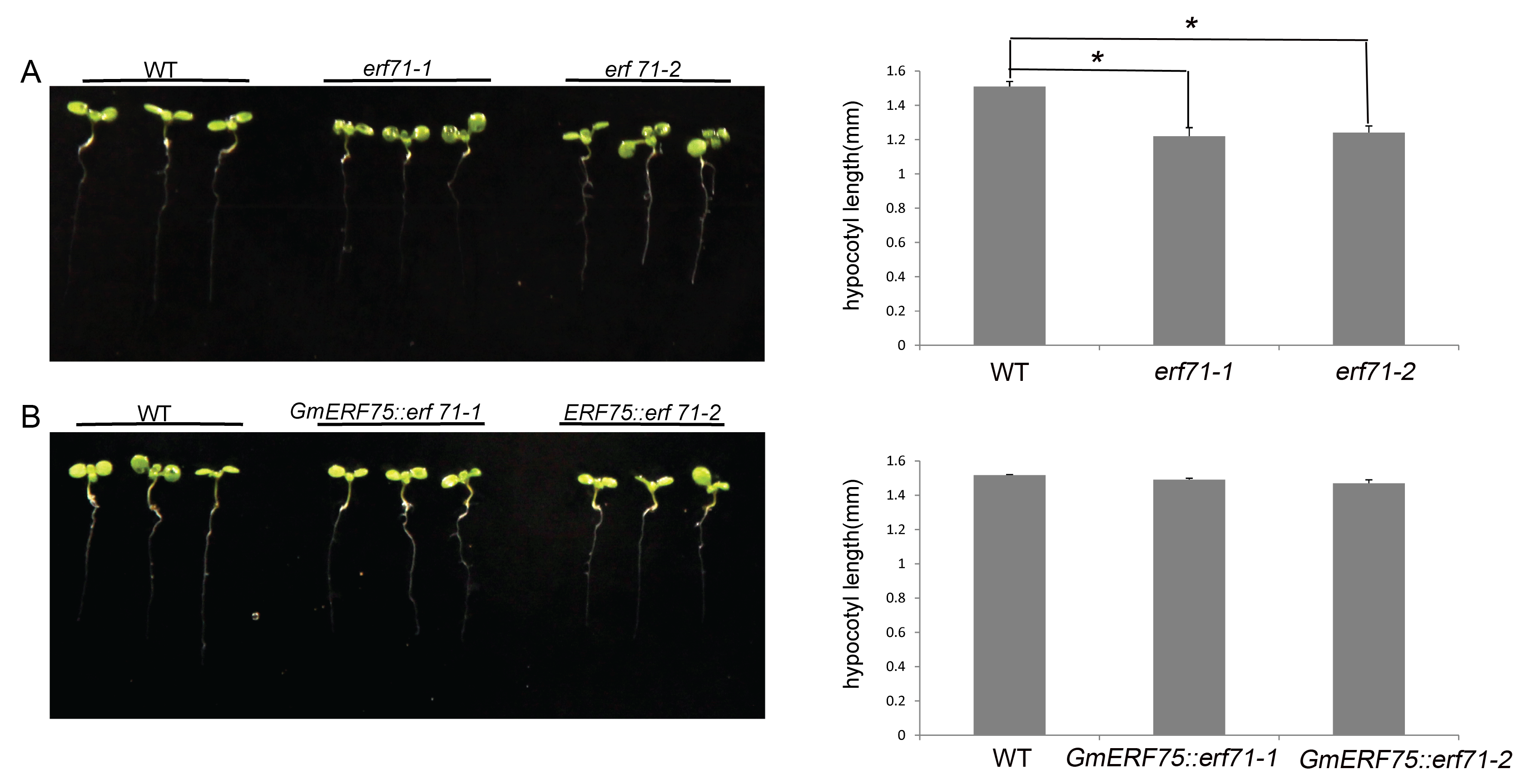

Supplement: Supplementary file 8 — Additional file 8: Figure S6. GmERF75 rescued the short hypocotyl length phenotype of two erf71 mutants. (A) The erf71 mutants displayed shorter hypocotyls than the WT. (B) Overexpression of GmERF75 in the mutants partially rescued the short hypocotyl length phenotype. The histogram on the right shows the distribution of hypocotyl lengths for at least 30 seedlings. [file 12870_2019_2066_MOESM8_ESM.tif]
